# Lymphome B diffus à grandes cellules du sinus éthmoïdal

**DOI:** 10.11604/pamj.2015.22.236.8228

**Published:** 2015-11-12

**Authors:** Rim Lahiani, Madiha Mahfoudhi

**Affiliations:** 1 Service d'ORL, Hôpital Charles Nicolle, Tunis, Tunisie; 2 Service de Médecine Interne A, Hôpital Charles Nicolle, Tunis, Tunisie

**Keywords:** Lymphome B, sinus éthmoïdal, immuno-histochimie, B lymphoma, ethmoid sinus, immunohistochemistry

## Image en medicine

Le lymphome diffus à grandes cellules est un lymphome malin non hodgkinien qui atteint rarement le sinus éthmoïdal. Le pronostic dépend du stade de découverte, de la rapidité du diagnostic et de la prise en charge thérapeutique. Patiente âgée de 64 ans a été admise pour exploration d'une céphalée récente associée à des douleurs oculaires gauches évoluant depuis un mois sans flou visuel ni baisse de l'acuité visuelle. L'examen physique n'a pas révélé de fièvre ni d'adénopathies périphériques. L'endoscopie nasale a trouvé une tumeur du sinus éthmoïdal gaucheinfiltrant la cavité nasale homolatérale. La TDM du massif facial a révélé une formation éthmoïdale gauche de densité tissulaire de 3 cm de grand axe avec amincissement de la paroi interne de l'orbite gauche. L'examen anatomo-pathologique d'une biopsie réalisée à travers la cavité nasale a montré des infiltrats diffus de grands lymphocytes atypiques avec des noyaux hyperchromatiques. L'étude immuno-histochimique a confirmé le diagnostic d'un lymphome Bdiffus à grandes cellules du sinus éthmoïdal. Le bilan d'extension du lymphome était négatif. Le traitement s'est basé sur une radiothérapie couplée à une chimiothérapie. L'évolution était marquée par l'absence d'extension ou de métastases avec un recul de 5 mois.

**Figure 1:**
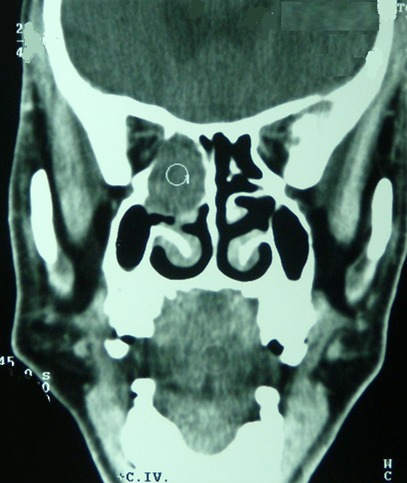
TDM du massif facial (coupe coronale): formation éthmoïdale gauche de densité tissulaire de 3 cm de grand axe avec amincissement de la paroi interne de l'orbite gauche

